# Supplementary material for: Evaluating the Potential Safety Risk of Plant-Based Meat Analogues by Analyzing Microbial Community Composition
Source: Foods. 2023 Dec 29;13(1):117. doi: 10.3390/foods13010117 (PMC10778452; doi:10.3390/foods13010117)
Supplement: Supplementary file 1 [file foods-13-00117-s001.zip › foods-2784105-supplementary.pdf]

**Table S1** Alpha diversity indexes calculated for the bacterial communities of per sample for high-throughput sequence reads determined at a 97% similarity

| Samples | Raw sequence | Average length(bp) | OTU number | Ace | Chao | Good's Coverage | Shannon | Simpson | Sobs |
|---|---|---|---|---|---|---|---|---|---|
| VS1 | 93211 | 426.867988 | 318 | 592.205686 | 479.052632 | 0.998004 | 1.496656 | 0.333159 | 318 |
| VS2 | 112304 | 407.349979 | 197 | 277.755788 | 264.857143 | 0.998885 | 1.105155 | 0.509463 | 197 |
| VS3 | 97553 | 417.921253 | 405 | 583.393472 | 545.096386 | 0.997755 | 1.739656 | 0.293471 | 405 |
| BJ1 | 82777 | 410.475168 | 234 | 542.757965 | 468.37037 | 0.998342 | 1.113601 | 0.444138 | 234 |
| BJ2 | 99454 | 413.107517 | 452 | 610.527015 | 592.411765 | 0.997725 | 1.490988 | 0.389327 | 452 |
| BJ3 | 84900 | 425.04371 | 367 | 762.495113 | 561.121622 | 0.997505 | 1.158849 | 0.430293 | 367 |
| CM1 | 97409 | 427.931813 | 910 | 937.324604 | 946.15873 | 0.999002 | 3.67362 | 0.124084 | 910 |
| CM2 | 102304 | 417.118558 | 607 | 770.033843 | 747.777778 | 0.997329 | 2.22359 | 0.247608 | 607 |
| CM3 | 88822 | 424.457556 | 938 | 987.590809 | 986.944882 | 0.998356 | 2.557575 | 0.229173 | 938 |
| BG1 | 75721 | 409.04094 | 660 | 885.39625 | 838.717949 | 0.996992 | 2.775596 | 0.184579 | 660 |
| BG2 | 94806 | 417.274698 | 366 | 572.483329 | 511.265823 | 0.997769 | 1.640771 | 0.383047 | 366 |
| BG3 | 86311 | 415.69742 | 424 | 594.72525 | 556.136364 | 0.997755 | 1.958958 | 0.275444 | 424 |
| BGB1 | 97580 | 406.441761 | 273 | 400.57854 | 353.815385 | 0.998488 | 0.993293 | 0.630178 | 273 |
| BGB2 | 89140 | 406.566065 | 684 | 777.311772 | 766.457627 | 0.997945 | 2.971983 | 0.12389 | 684 |
| BGB3 | 95336 | 422.462805 | 1166 | 1253.361982 | 1248.118644 | 0.997491 | 3.871893 | 0.087487 | 1166 |

**Table S2** Alpha diversity indexes calculated for the fungal communities of per sample for high-throughput sequence reads determined at a 97% similarity

| Samples | Raw sequence | Average length(bp) | OTU number | Ace | Chao | Good's Coverage | Shannon | Simpson | Sobs |
|---|---|---|---|---|---|---|---|---|---|
| VS1 | 84621 | 382.722433 | 66 | 93.10244 | 96.66667 | 0.99962 | 0.606344 | 0.647136 | 66 |
| VS2 | 97444 | 382.626996 | 82 | 140.0334 | 129.25 | 0.999557 | 0.806157 | 0.530209 | 82 |
| VS3 | 89674 | 382.81048 | 67 | 74.88906 | 72.07692 | 0.99981 | 0.512774 | 0.751249 | 67 |
| BJ1 | 100257 | 381.702175 | 201 | 210.7823 | 210 | 0.999715 | 3.103253 | 0.101142 | 201 |
| BJ2 | 104283 | 381.728546 | 203 | 214.3806 | 212 | 0.999699 | 3.136599 | 0.088174 | 203 |
| BJ3 | 102220 | 381.558961 | 216 | 227.2058 | 228.6667 | 0.999683 | 3.030481 | 0.11606 | 216 |
| CM1 | 79115 | 381.338204 | 58 | 65.42568 | 65.5 | 0.999842 | 0.570838 | 0.745955 | 58 |
| CM2 | 82622 | 382.937825 | 59 | 65.58466 | 65.42857 | 0.999842 | 0.629044 | 0.689422 | 59 |
| CM3 | 90087 | 382.609611 | 61 | 68.54312 | 70.16667 | 0.999826 | 0.689127 | 0.754127 | 61 |
| BG1 | 105603 | 382.222939 | 235 | 253.092 | 254.3333 | 0.999541 | 2.926258 | 0.136472 | 235 |
| BG2 | 105335 | 382.182124 | 239 | 258.5875 | 255.9167 | 0.999541 | 3.127979 | 0.10844 | 239 |
| BG3 | 105355 | 382.244934 | 244 | 256.7931 | 255 | 0.999636 | 3.207432 | 0.100338 | 244 |
| BGB1 | 95870 | 381.503275 | 194 | 215.1289 | 223.1765 | 0.999493 | 2.203012 | 0.302352 | 194 |
| BGB2 | 96199 | 381.476481 | 200 | 211.3123 | 208.0769 | 0.999667 | 2.330416 | 0.275595 | 200 |
| BGB3 | 104772 | 381.371464 | 175 | 190.2169 | 188.5882 | 0.999652 | 2.173583 | 0.323474 | 175 |

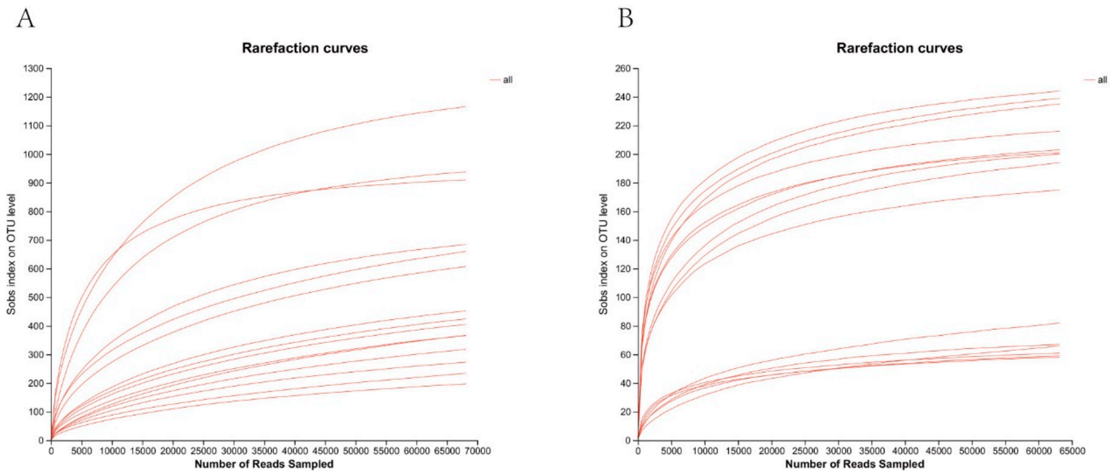


**Figure S1.** Rarefaction curves of the samples in analyzing the microbial communities. (A) Rarefaction curves for bacterial community; (B) Rarefaction curves for fungal community;
